# Multi-cohort screening of PANoptosis-related genes reveals PARP1 as a key target, and AZD5305 exerts anti-tumor effects in neuroblastoma

**DOI:** 10.3389/fonc.2026.1903085

**Published:** 2026-10-12

**Authors:** Shujun Shi, Zhaoxia Zhang, Junyi Luo, Zhaoying Wang, Tao Mi, Liming Jin, Xiangpan Kong, Jinkui Wang, Junhong Liu, Li Wang, Dawei He

**Affiliations:** 1 Department of Urology, Children’s Hospital of Chongqing Medical University, National Clinical Research Center for Child Health and Disorders, the Laboratory of Targeted Delivery of Traditional Chinese Medicine, Chongqing, China; 2 Department of anesthesiology, Children’s Hospital of Chongqing Medical University, Chongqing, China; 3 The Second Affiliated Hospital of Chongqing Medical University, Key Laboratory of Integrated Therapy of Traditional Chinese Medicine for Tumors, Chongqing Municipal Administration of Traditional Chinese Medicine, Chongqing, China; 4 Department of Pediatrics, The Second Clinical College of Chongqing Medical University, The Second Affiliated Hospital of Chongqing Medical University, Chongqing, China

**Keywords:** AZD5305, neuroblastoma, PANoptosis, prognosis, tumour immunity

## Abstract

**Background:**

Neuroblastoma (NB) is the most prevalent extracranial solid malignant tumor in infants and children. Approximately half of children with high-risk NB exhibit primary resistance to first-line therapy or experience disease relapse within two years post-treatment, leading to dismal overall survival. PANoptosis is an inflammatory programmed cell death modality integrating pyroptosis, apoptosis and necroptosis; nevertheless, the functional relevance of PANoptosis-related genes (PRGs) in NB remains largely undefined. This study aimed to explore PRGs associated with NB prognosis and their link to the tumor immune microenvironment, so as to identify novel therapeutic targets for NB.

**Methods:**

mRNA expression profiles from three public NB datasets (GSE49711-NB, TARGET-NB and E-MTAB-8248-NB) were used to investigate the prognostic roles of 19 previously reported PRGs. Independent Cox regression screening was performed in each cohort to identify cohort-specific prognostic PRGs, followed by Venn intersection analysis to identify conserved prognostic genes. PARP1 expression was validated in tumor tissues from 127 clinical NB patients. The GIS score based on PARP1 expression and clinical metadata were combined to assess its prognostic value. *In vitro* functional assays were performed to explore the anti-tumor effects and mechanisms of the PARP1 inhibitor AZD5305.

**Results:**

Venn intersection analysis identified PARP1 as the only shared gene with consistent prognostic significance across heterogeneous NB patient populations. *In vitro* experiments revealed that AZD5305 markedly suppressed NB cell proliferation, migration and invasion, triggered cell apoptosis, and arrested cell cycle progression at the G0/G1 phase.

**Conclusion:**

This study adopts a multi-cohort parallel screening strategy to mine conserved therapeutic targets for NB. Our data indicate that PARP1 inhibition via AZD5305 represents a promising therapeutic strategy for NB treatment.

## Introduction

Neuroblastoma (NB), the most common extracranial solid malignancy in infancy and childhood, is an embryonic tumour originating from primitive sympathetic precursor cells (1). In the world, the incidence of NB accounts for 6% to 10% of all malignant tumours in children, ranking first in the incidence of malignant solid tumours in children in China (2). The clinical manifestations of NB patients with different ages, tumour locations, and degrees of tissue differentiation are very different, mainly invasive growth with unclear boundaries, which is difficult to remove by surgery. At present, NB is mainly treated with a comprehensive treatment mode of surgery, radiotherapy, and chemotherapy. Although specific progress has been made, NB is usually progressive and highly malignant, and nearly half of high-risk NB children are insensitive to first-line treatment or relapse within the first 2 years after treatment. Therefore, the prognosis of high-risk NB children is still inferior, and it has been reported that the long-term survival rate of children with advanced and metastatic disease is<40% (3, 4). NB has become a significant health problem and clinical treatment problem that seriously endangers children’s health. Therefore, it is still one of the most urgent problems to find specific therapeutic targets for NB and improve the survival rate of children with NB, especially high-risk disease. Cell death is the centre of development, body homeostasis, and immune response, among which pyroptosis, apoptosis, and necrotic apoptosis are the most clearly defined programmed cell death pathways (PCD) in genetics (5). Previous studies have reported that multiple cell death phenomena also exist in tumours, and a large amount of evidence has shown that PCD subroutines (including apoptosis, pyrosinecrotic apoptosis, and siderosis) play a key role in tumorigenesis. Loss of control over a single or combined PCD subroutine may lead to the development of a tumour (6). Earlier studies highlighted the unique regulation of each pathway, but emerging research has uncovered co-regulation and interactions between these seemingly distinct cellule-death complexes, hence the concept of PANoptosis. PANoptosis is an inflammatory programmed cell death regulated by the PANoptosome (PANoptotic body) complex with critical features of pyroptosis, apoptosis, and necroptosis (7). Abnormal expression of PANoptosis-related genes (PRGs) has been observed in various tumour types, such as NLRP3, caspase-8, and TNFAIP3, and significant mutations have been detected. It is important to note that many PRGs are tumour risk factors for different tumour types. For example, Zhang et al. found high positive expression of CD8A and CXCL6 in both LIHC tissues and most human HCC cell lines, suggesting that both PRGs may be potential biomarkers for diagnosing and treating hepatocellular carcinoma (8). Wang et al. found that PRGs were strongly associated with tumour progression in patients with cutaneous melanoma. PRGs can be used not only for risk assessment and OS prediction in patients with cutaneous melanoma but also to reconstruct the immune landscape of patients with cutaneous melanoma (9). Wang et al. found that LGR5 and VSIG4 were differentially expressed in normal colon tissue and colon cancer samples, demonstrating the potential of PANoptosis-based molecular clustering and prognostic features in predicting survival and tumour microenvironment (TME) in colon cancer patients (10). PRGs and PANoptosis scores have been reported to be significantly associated with survival in patients with 21 and 14 tumour types, respectively (11). Inhibition of the interaction of PRGs, such as ZBP1 and RIPK3, can promote tumorigenesis (12). These findings suggest that PANoptosis may positively affect certain tumour types, and induction of PANoptosis may help inhibit the development and progression of tumours. These findings suggest that cancer progression is related to mechanisms that allow cancer cells to bypass PANoptosis. Therefore, understanding the link between PANoptosis and cancer may provide deeper insights into the occurrence and treatment of cancer and explore new therapeutic strategies by interfering with the escape mechanism of PANoptosis in cancer cells (13). Some studies have reported the existence of pro-death in NB, and Zhao et al. found that the sensitivity of NB to cisplatin could be inhibited by inhibiting pro-death (14). Li et al. found that NB with high pyrodeath showed a reasonable overall survival rate and more anti-tumor immune cell infiltration (15). Luo et al. integrated four NB datasets to construct a new scoring method, called focal score, to quantify the level of pyrogenic apoptosis in NB; they found that a high focal score was associated with good prognosis in NB patients, immune activation and increased response to checkpoint blocking immunotherapy (16). Failure of apoptosis is thought to contribute to tumorigenesis because it regulates the balance between cell proliferation and death (17). In addition, failure to experience apoptosis may lead to treatment resistance, as the cytotoxic activity of commonly used clinical anticancer therapies (e.g chemotherapy, gamma ray irradiation, or immunotherapy) is mediated primarily by triggering apoptosis of tumour cells (18). Necrotic apoptosis is morphologically necrotic but induced by the same stimuli as apoptosis (19). A study by B J Maurer et al. demonstrated that synthetic retinoid N-(4-hydroxyphenyl) retinamide (4-HPR or fenvistamine) could be a potential strategy for NB treatment by inducing necrosis of NB cells (20). However, no bioinformatics-based mechanisms related to PANoptosis have been identified in NB. This study aims to screen stable PANoptosis-related therapeutic targets for NB via parallel prognostic analysis of three independent patient cohorts, rather than establish and validate a universal predictive gene signature to serve as potential therapeutic targets for NB.

## Materials and methods

### Data sources and processing for NB

We retrieved three independent neuroblastoma cohorts (GSE49711, Target-NB, E-MTAB-8248) from public databases (data access date: July 27, 2023) for parallel prognostic gene screening. All patients with incomplete follow-up records were excluded, leaving a total of 968 individuals included in subsequent survival analysis. Collected clinical covariates comprised age, sex, COG risk stratification, MYCN amplification status, INSS stage, and overall survival status.

This multi-cohort parallel analytical framework was designed to identify PANoptosis-related genes (PRGs) with consistent prognostic performance across heterogeneous patient populations, rather than to train and externally validate a single unified predictive gene signature. Independent survival regression analyses were performed within each cohort, and PARP1 was finally identified as the only PRG significantly associated with prognosis in all three datasets.

All datasets used in this study are publicly accessible open resources; therefore, additional ethical approval or patient informed consent was not required. This research fully complies with the data release and publication specifications of the GEO, TARGET and ArrayExpress databases.

### Consensus cluster analysis of PRGs

Nineteen PRGs were retrieved from recent publications (10, 21). Unsupervised cluster analysis was used to classify 498 NB patients in GSE49711 according to the expression profiles of 19 PRGs. Cluster analysis was performed using ConsensusClusterPlus (ConsensusClusterPlus: a class discovery tool with confidence assessment and item tracking) (22), clustering was performed using clustering pam with 1-pearson correlation distance And resampling 80% of the sample 10 times repetitively. The optimal number of clusters was determined using the experience cumulative distribution function graph. We used the R package stats (version 3.6.0) for analysis for PCA. Specifically, we first performed a z-score on the expression profile and used the prcomp function for dimensionality reduction analysis to obtain the matrix after dimensionality reduction. Next, we evaluated the correlation between the 19 PRGs, clinicopathological features, and prognosis to determine the clinical utility of both subtypes C1 and C2 established by consensus clustering. Kaplan-Meier methods and log-rank tests were used to compare outcomes between the two groups. To explore the differences in biological processes between the two PRG clusters, we used the R software package to calculate the enrichment score in gene sets for each sample from GSVA (GSVA, version 1.40.1). We predefined the gene row rank. Specifically, we first used gene expression profile, Using Hanzelmann et al.’s method (23) and from the Signatures Database (http://www.gsea-msigdb.org/gsea/downloads.jsp) To download the c2.Cp.Kegg.V7.4.Symbols. The GMT subsets (24) evaluate the relevant pathways and molecular mechanisms. The R package “limma” is used to analyze differences in functional annotation. A significant change in the KEGG pathway was defined as a P-value less than 0.05 (25).We used the R-package “IOBR” to estimate the matrix and immune scores and the degree of TME-associated cell invasion in NB tumour tissue (26). We then used the two-sample Wilcoxon test to compare the differences in infiltrated immune cells in the C1 and C2 subtypes and the high-risk and low-risk subgroups. In assessing the association between the C1 and C2 subtypes and TME, we analyzed the association between the abundance of immune cells and these 19 PRGs. The abundance of immune cell infiltration was compared between the C1 and C2 subtypes and between the high-risk and low-risk groups. For detailed analysis methods, see previous work (27).

### Construction of PANoptosis-related prognostic features

It should be clarified that the primary goal of this multi-cohort analysis was not to build and externally validate a fixed predictive signature. Instead, we performed independent prognostic Cox regression screening within each of the three separate cohorts to identify PRGs with stable prognostic relevance across diverse NB patients, and intersected candidate genes from all cohorts to screen universal therapeutic targets.

The R package glmnet was used to integrate survival time, survival status and PRG expression matrices. LASSO regression coupled with 10-fold cross-validation was first conducted to filter candidate genes, after which multivariate Cox regression (via the survival package) was applied to select PRGs with independent prognostic significance exclusively in the GSE49711 cohort.

After calculating continuous RiskScore for each patient, maximally selected rank statistics implemented in the R package maxstat (v0.7-25) was used to determine the optimal cutoff for risk stratification. To eliminate statistical bias caused by extremely unbalanced subgroups, we limited the sample proportion of each subgroup to 25%–75% of the total cohort. The algorithm scanned all possible RiskScore values and selected the threshold with the largest inter-group survival difference, which was calculated as 43.472. Patients were divided into high-risk and low-risk subgroups accordingly. Kaplan–Meier curves and the Log-rank test were adopted to compare overall survival between the two subgroups (P = 3.4×10^-35^). The identical maxstat stratification workflow with the same sample proportion constraint was reproduced in the Target-NB and E-MTAB-8248 cohorts to maintain consistent analytical standards across all datasets.

Within the GSE49711 cohort, univariate and multivariate Cox regression analyses integrating RiskScore and multiple clinicopathological variables were further performed to screen independent prognostic factors. Separate multivariate Cox regression models were also built for the Target-NB and E-MTAB-8248 cohorts to extract their respective prognosis-associated PRGs. Kaplan–Meier analysis was adopted to assess survival differences between the two PANoptosis molecular subtypes (C1 and C2). Time-dependent receiver operating characteristic (ROC) curves and corresponding area under the curve (AUC) values were plotted to quantify the predictive capacity of the RiskScore signature built solely on the GSE49711 dataset.

### Screening of key PRGs in 3 databases

The prognostic-associated PRGs selected from three independent cohorts were screened for critical PRGs associated with prognosis. The clinicopathological information in the GSE49711-NB cohort was combined to analyze further the correlation of critical PRG genes with COG risk stratification and clinicopathological variables such as MYCN amplification status.

### Immunohistochemical staining

The Department of Pathology, Children’s Hospital, Affiliated with Chongqing Medical University, provided the tissue sections. Paraffin sections of tumour tissue were routinely dewaxed, then repaired with microwave, sealed with 0.5%bovine serum albumin (Sorabio, China), and incubated at 4 °C for primary antibody overnight. Washed 3 times with PBS, the corresponding secondary antibody was incubated at room temperature (1:200 dilution, China) for one h. Positive staining was obtained by incubation with 3, 3’-diaminobenzidine (Zhongshan, China). Sections were then reverse-stained with hematoxylin. The stained slides were then scanned using the TEKSQRAY slide scanning Imaging system (Sheng et al., China), and the slides were evaluated for PARP1 expression by experienced pathologists. Five high-power fields (×400) were randomly observed on each slice, and an average was obtained; the scoring standard was German chemical score (GIS) (28): (1) staining intensity: cells not coloured, score 0; Light yellow, one score; Medium yellow or brown, 2 points; Dark brown or tan,3; (2) Proportion of positive cells: no positive cells, 0 points; <25%, 1 points; <50%, 2 points; <75%, 3 points; Greater than 75%, 4 points. Finally, add the two component scores: 0-3, which are low expressions, and Score 4-7, which are high expressions.

### Clinical data statistics

Our retrospective study included primary retroperitoneal neuroblastoma children who underwent surgical management at the Children’s Hospital Affiliated with Chongqing Medical University from 2017 to 2023. This study was approved by Insitutional review Board of Children’s Hospital of Chongqing Medical University (2022) Lun Review (Research) No.170. Exclusion criteria were no surgical treatment, patient consent, and insufficient tumour tissue. 127 patients met the inclusion criteria. We collected the patient’s medical record number, age, gender, INSS stage, COG risk grouping, MYCN amplification status and other information. We followed up on the survival status and survival time of the patients. Detailed data on these patients were presented in **Supplementary Table 1**. Statistical analysis was performed using IBM SPSS 25.0 window Statistics (SPSS Statistics, Statistics 25, IBM Corporation, Armonk, NY, USA). Survival datawere analyzed by multivariate Cox regression, and then established a nomogram by Cox method to further predict the survival of NB patients in the GSE49711-NB cohort. C-index, AUC and DCA were used to evaluate the model. P ≤ 0.05 was considered statistically significant.

### Animal experiments and histopathological studies

BALB/c mice were purchased from SPF Biotechnology (China, Beijing) andare raised under specific pathogen-free (SPF) conditions. This study adhered to international animal ethical research guidelines and received approval from the Ethics Committee of Chongqing Medical University Children’s Hospital (Approval No.: CHCMU-IACUC20220323001). One million Neuro-2a cells were subcutaneously injected into the right axilla of 3-week-old male mice. When the tumor volume reached 90–100 mm³, the mice were randomly divided into six groups: control group, low-dose group (1 mg/kg), high-dose group (2 mg/kg), cisplatin-positive control group (5 mg/kg cisplatin), and combination therapy group (2 mg/kg AZD5305 + 5 mg/kg cisplatin or 2 mg/kg AZD5305 + 2.5 mg/kg cisplatin), with six mice in each group. Mice received daily intravenous injections of AZD5305 for seven days, while cisplatin was administered via intraperitoneal injection every other day for a total of three doses; the control group received only saline. Body weight and tumor volume were measured daily (tumor volume = length × short side²/2). At the end of the experiment, blood, subcutaneous tumor tissues, and major organs (heart, liver, kidneys, lungs, and spleen) were collected from the mice. Each tissue sample was stored in liquid nitrogen and 4% polyformaldehyde (Beyotime, product code P0099, China Shanghai). Blood samples were obtained from the orbit, while serum samples were isolated by the Laboratory Department of Chongqing Medical University Children’s Hospital for liver and kidney function tests (including ALT, AST, Cr, and BUN).

Tissue samples fixed with 4% polyformaldehyde were embedded in paraffin and prepared into 4 µm thick sections. The sections underwent dewaxing and rehydration, followed by HE staining using hematoxylin, eosin, different concentrations of alcohol, and xylene in sequence. Kidney tissues were stained with the Masson’s Trichrome Stain Kit(CAS No:G1340,Beijing Solarbio Science & Technology Co., Ltd.). All images were observed and acquired using an Eclipse Ci microscope (Nikon, Tokyo, Japan).

### Cell lines and cell culture

The SK-N-SH cell line was purchased from the Chinese Academy of Sciences Cell Library, while SK-N-DZ cell line was obtained from the American Association for Clinical and Research Laboratories (ATCC) in Manassas, Virginia. Cells were cultured routinely in DMEM medium containing 10% FBS, with experimental conditions following previous studies (29). After achieving cell adhesion, the PARP1 selective inhibitor saruparib (AZD5305) (MCE, CAS No 2589531-76-8, Monmouth Junction, NJ, USA) were administered to complete functional assays.

### Cell proliferation activity assay

Cell proliferation activity was detected using the CCK-8 Assay Kit (Chongqing BGBiotechnology Co., Ltd., BG0025). SK-N-SH and SK-N-DZ cells were inoculated into 96-well plates. After complete culture for 12 hours, the medium was replaced with complete culture medium containing different concentrations of AZD5303, followed by incubation for 48 hours. The absorbance at 450 nm wavelength was measured after 2 hours of treatment to calculate half inhibitory concentration of AZD5303. Subsequently, low, medium, and high concentrations (1/2 IC50, IC50, and 2 IC50) of AZD5303 were applied to SK-N-SH and SK-N-DZ cells. CCK-8 absorbance was measured at 24,48, and 72 hours to determine the inhibition rate of proliferation. Additionally, cell viability was assessed using the Edu Assay Kit. After culturing SK-N-SH and SK-N-DZ cells in 24-well plates for 12 hours, the medium was replaced with low, medium, and high concentration AZD5305 complete culture medium for 48 hours. The cells treated with AZD5305 were labeled using the Edu Working Solution in the Edu Assay Kit (Beyotime Biotechnology, C0071S, China). Cells were fixed with 4% polyformaldehyde, then co-incubated with Click Reaction Liquid for 2 hours (37 °C, dark) before nuclear staining. All images were captured using the ECLIPSE 90i fluorescence microscope.

### Cell migration and invasion ability detection

SK-N-SH and SK-N-DZ cells were cultured in 6-well plates until complete cell fusion, after which scratch lines were created. The cultures were maintained in serum-free medium containing varying concentrations of AZD5305, with the scratch areas recorded using an ECLIPSE microscope (Nikon, Japan) at 0, 24, and 48 hours post-treatment. Both cell lines were seeded into Transwell chambers (FALCON, 353097, Newport, Tennessee, USA) pre-coated with matrix gel, with different concentrations of AZD5303 added to both groups. After 48-hour treatment, the chambers were removed, fixed with 4% polyformaldehyde, and stained with crystal violet solution (Beyotime Biotechnology, C0071S, China). Optical microscopic images were captured and cell counts were quantified using ImageJ software (version 1.52).

### Apoptosis and cell cycle analysis

The Annexin V apoptosis detection kit (Elabscience Biotechnology Co., Ltd., E-CK-A218,China), was employed for flow cytometric analysis. SK-N-SH and SK-N-DZ cells were cultured under standard conditions for 12 hours, then maintained in medium containing varying concentrations of AZD5303 for an additional 48 hours. Following the protocol instructions, Annexin V detection reagent was added, and the apoptosis cell percentage was quantified using a flow cytometer (BD Pharmingen, USA). The data were subsequently analyzed with FlowJo software version 10.8.1. The cell cycle was detected using propidium iodide (PI)/RNase staining buffer (Beyotime Biotechnology, C1052, China). After 12-hour routine culture of SK-N-SH and SK-N-DZ cells, the medium containing different concentrations of AZD5303 was replaced and cultured for an additional 48 hours. Cell cycle detection reagents were added according to the manual instructions, followed by flow cytometry analysis of cell cycles. Data were analyzed using ModFit LT software (version 3.2).

### Statistical analysis

All bioinformatic analyses and R packages were performed using R software. Statistical analysis of experimental data was completed by GraphPad 8.0 software. The comparison between the two groups was t-test, and the comparison between multiple groups was one-way ANOVA. P<0.05 indicated the significant difference in statistics. P<0.05 indicated statistically significant differences. All experiments were repeated three times.

## Results

### Molecular subtypes defined by PANoptosis-related genes in the GSE49711 cohort

We first analyzed the expression landscape of 19 well-documented PRGs across the GSE49711 NB cohort. Consensus clustering was applied to categorize patients into two distinct PANoptosis-associated molecular subtypes. Significant disparities in overall survival rates, stromal/immune scores and immune cell infiltration abundance were detected between the two subtypes, and all clustering-related analyses and visualization results were archived in **Supplementary Figure 1**. Given that subtype classification was merely an exploratory single-cohort analysis without cross-cohort reproducibility, we did not expand subsequent prognostic or immunological validations based on this clustering system.

### Screening of prognostic PRGs in three independent NB cohorts

We separately identified 11, 5 and 6 prognosis-associated PRGs from the GSE49711, Target-NB and E-MTAB-8248 cohorts through independent cohort-wise Cox screening, respectively. LASSO-Cox regression was conducted in the GSE49711 cohort to preliminarily screen 11 prognosis-linked PRGs for intra-cohort risk evaluation. Instead of validating this 11-gene signature externally. First, lasso Cox regression analysis was performed to determine the optimal value of the penalty parameters and establish the optimal prognostic features. The logI sequence generated a coefficient profile to obtain 16 non-zero coefficients (**Figure 1A**). Finally, a PANoptosis-related model of 16 genes with optimal predictive efficiency was obtained. Subsequently, the 16 PRGs genes were subjected to multivariate Cox multivariate analysis to screen out 11 PRGs associated with NB prognosis (**Figure 1B**), and the RiskScore of patients was obtained. This signature was only used for survival analysis within the GSE49711 cohort. According to the best cutoff value of RiskScore 43.472, the patients were divided into high-risk and low-risk groups. Each patient’s gene expression of 11 characteristic PRGs was visualized using heat maps (**Figure 1C**). A time-dependent ROC curve was used to assess the prediction accuracy of gene features associated with extrusion, with the area under the curve (AUC) values of 0.86 (0.94-0.78), 0.88 (0.92-0.83), and 0.89 (0.92-0.85) for predicting 1, 3, and 5-year survival, respectively (**Figure 1D**). Finally, as expected, in the GSE49711-NB cohort, NB patients in the low RiskScore subgroup had significantly better survival than those in the high RiskScore subgroup (**Figure 1E**).

**Figure 1:**
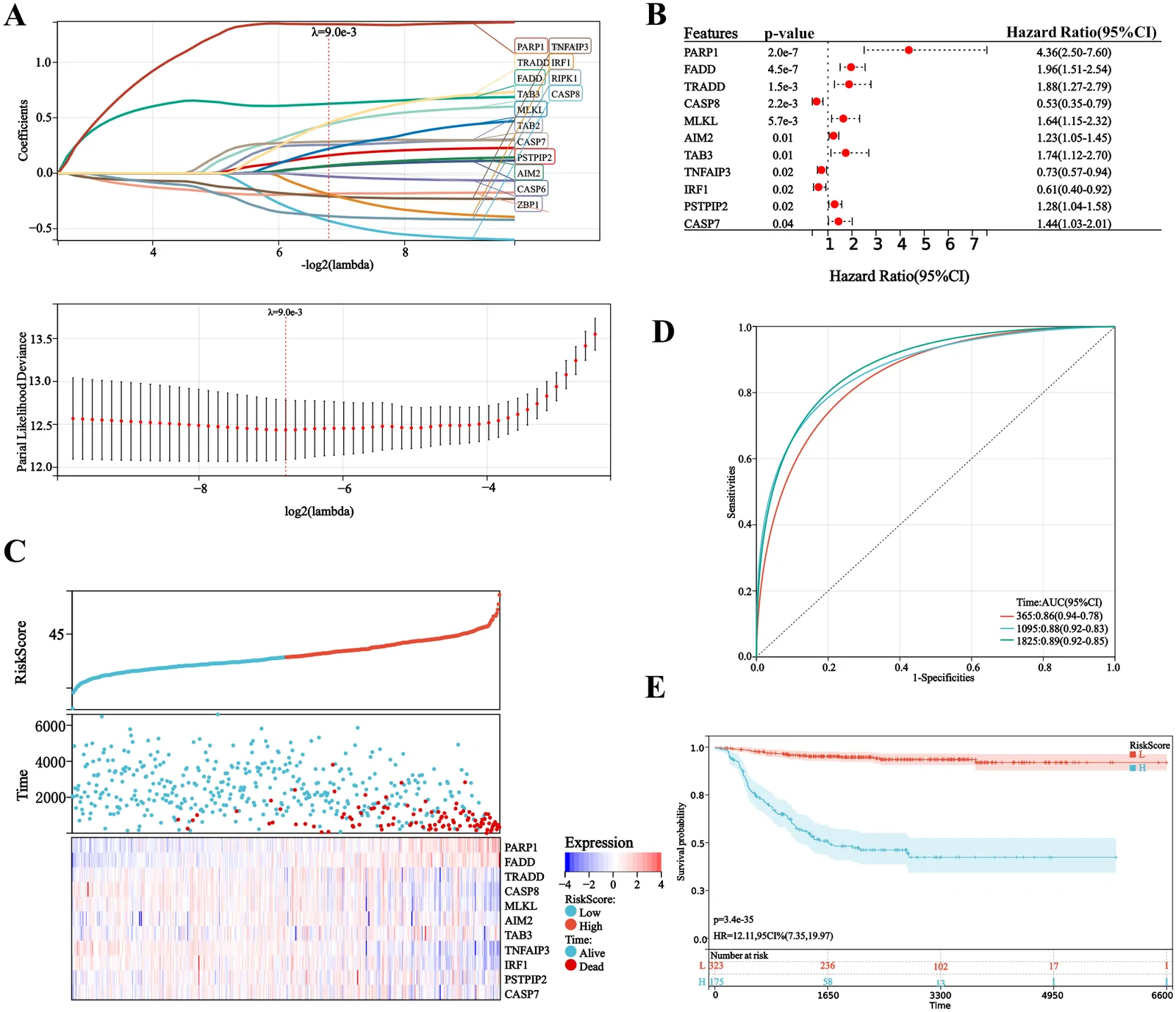
Construction of NB risk models based on PRGs prognostic features in the GSE-NB cohort(The 11-PRG prognostic signature constructed solely based on the GSE49711 cohort for intra-cohort survival stratification). **(A)** Lasso Cox regression analysis obtained a PRGs 16-gene model with optimal predictive efficiency. **(B)** 11 PRGs associated with the prognosis of NB were screened by Cox multifactor analysis. **(C)** Patients were divided into high and low based on PRGs risk scores for 11 prognostic feature In the risk group,the gene expression of each patient’s 11 characteristic PRGs was visualized using heat maps. **(D)** ROC curve of the 11 prognostic features of the NB risk model constructed by PRGs. **(E)** Survival differences between high-risk and low-risk groups(grouped according to PRGs risk scores for 11 prognostic characteristics) in the GSE-NB cohort.

We carried out separate univariate and multivariate Cox regression analyses in Target-NB and E-MTAB-8248-NB cohorts to screen independent prognostic PRGs for each dataset. In brief, 5 prognosis-relevant PRGs were identified in the Target-NB cohort, Independent multivariate Cox regression was performed on the Target-NB cohort to screen its own prognosis-related PANoptosis-related genes (PRGs), and five candidate genes (PARP1, TAB2, MLKL, ZBP1, RIPK3) were obtained (**Figure 2A**). The maxstat algorithm with consistent 25%–75% sample proportion constraints was used to calculate an independent optimal risk score cutoff of 3.24 specific to the Target-NB cohort, which was only applied for intra-cohort survival stratification. Kaplan–Meier analysis revealed markedly poorer overall survival for high-risk patients relative to low-risk individuals (**Figure 2B**). Heatmaps were plotted to visualize the expression profiles of the five cohort-specific prognostic PRGs across all Target-NB participants (**Figure 2C**). Time-dependent AUC values for 1-, 3-, and 5-year overall survival (OS) within this cohort were calculated as 0.73, 0.68 and 0.68, respectively (**Figure 2D**). While 6 prognostic PRGs were screened from the E-MTAB-8248-NB cohort. The identical independent Cox regression screening workflow was adopted for the E-MTAB-8248-NB cohort to extract its unique set of prognostic PRGs, including PARP1, RIPK1, GSDMD3, CASP7, FADD and NLRP3 (**Figure 2E**). A cohort-specific optimal cutoff value of 36.45 was separately calculated via the maxstat approach for internal risk grouping. Kaplan–Meier curves demonstrated significantly inferior survival outcomes in the high-risk subgroup (**Figure 2F**). Heatmaps were generated to display the expression patterns of the six prognostic PRGs unique to this cohort (**Figure 2G**). The time-dependent AUC values for predicting 1-, 3-, and 5-year OS in the E-MTAB-8248 cohort were 0.79, 0.83 and 0.85, correspondingly (**Figure 2H**).

**Figure 2:**
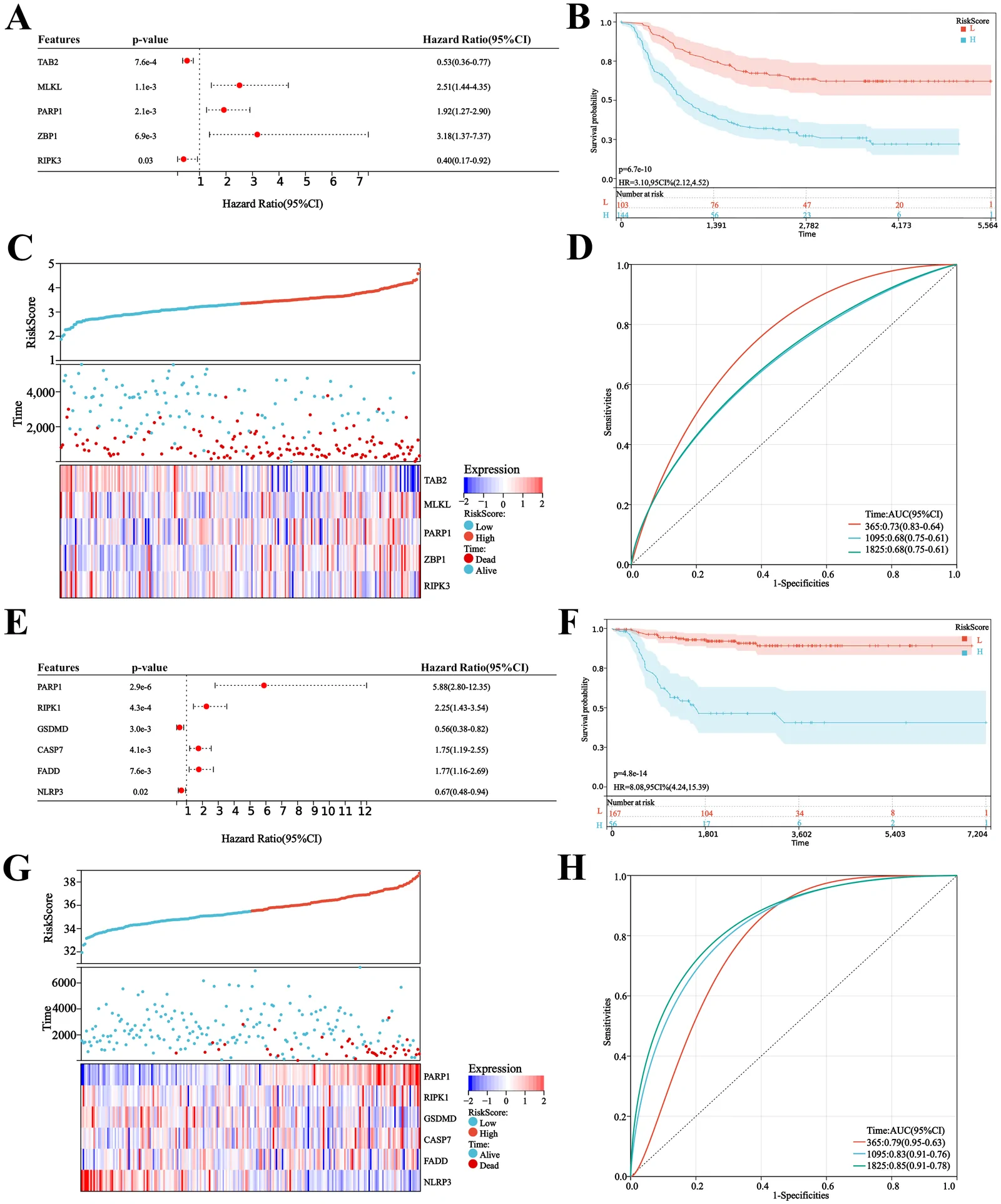
Independent multivariate Cox regression was performed in the Target-NB/E-MTAB-8248 cohort to screen prognosis-related PRGs separately. **(A)** Forest map of prognostic related PRGs in the Target-NB cohort(multivariate COX regression analysis). **(B)** KM curves of risk scores for the genes associated with the prognosis of NB in the Target-NB cohort dividing the high and low risk groups. **(C)** Heat maps of expression of 5 PRGs associated with prognosis for each patient in the Target-NB cohort. **(D)** ROC curves predicting 1-,3-,and 5-year survival ofNB patients in the Target-NB cohort. **(E)** Forest map of prognostic related PRGs in the E-MTAB-8248-NB cohort(multivariate COX regression analysis). **(F)** KM curve of risk scores of PRGs associated with prognosis of NB in the E-MTAB-8248-NB cohort dividing high and low risk groups. **(G)** Heat map of expression of 6 PRGs associated with prognosis in each patient in the E-MTAB-8248-NB cohort. **(H)** ROC curves predicting 1-year,3-year,and 5-year survival ofNB patients in the E-MTAB-8248-NB cohort.

### Clinical validation of PARP1 prognostic value in 127 NB patient specimens

The intersection of prognostic gene lists from the three cohorts yielded PARP1, the only gene with stable prognostic implication across heterogeneous NB patient populations (**Figure 3A**). Further correlation analysis based on GSE49711 clinical metadata illustrated that PARP1 expression was significantly elevated in deceased patients compared with survivors. Moreover, PARP1 expression levels were tightly linked to COG risk stratification and MYCN amplification status: higher PARP1 expression was observed in COG high-risk patients and individuals with MYCN amplification (**Figure 3B**).

**Figure 3:**
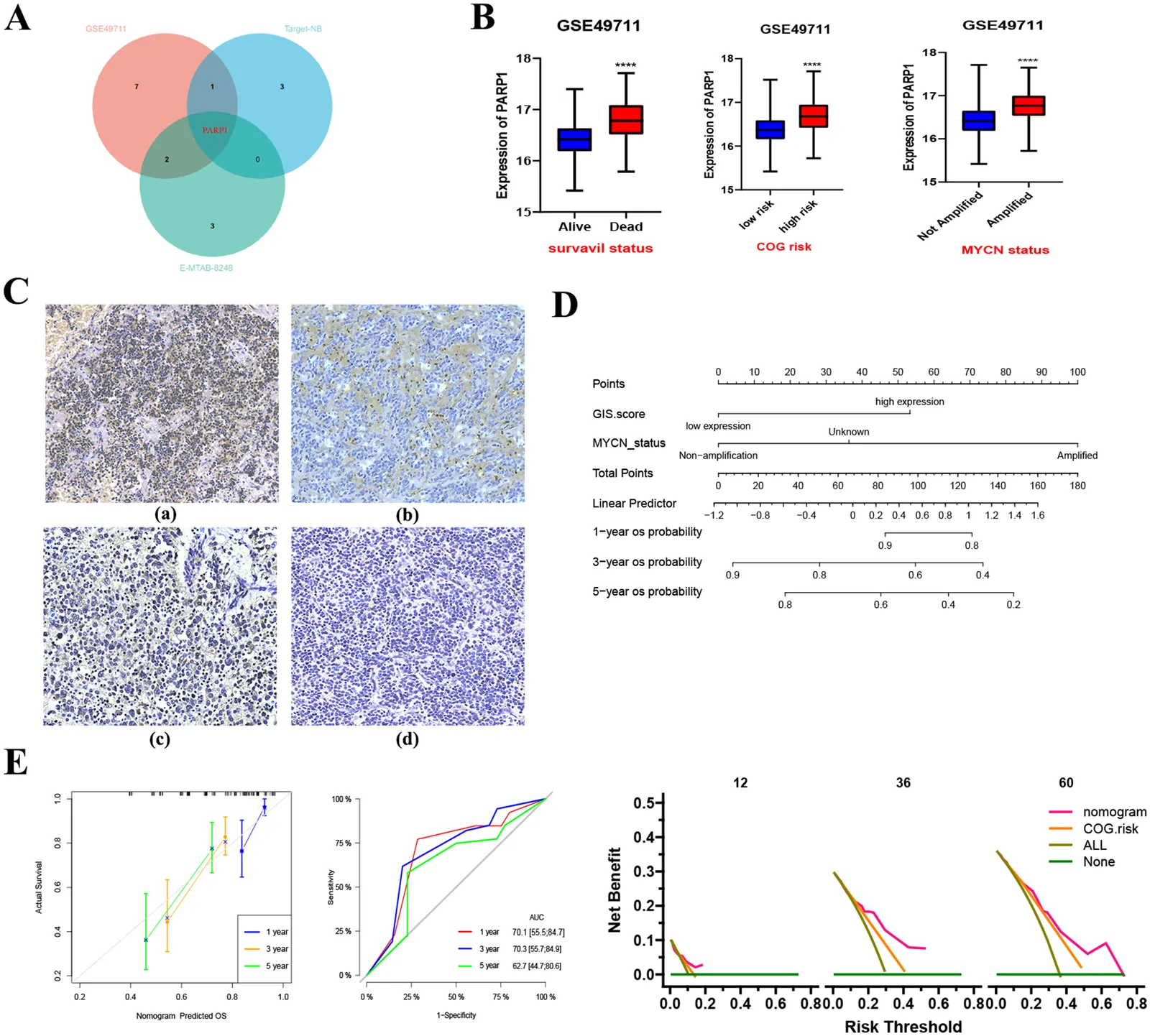
PARP1 gene screening and analysis of PARP1 expression levels(different survival states,COG risk groups,and MYCN amplification status) and Nomogram for predicting the prognosis of children with neuroblastoma combined with PARP1 expression and clinical information(data of children with neuroblastoma in our hospital). **(A)** Venn diagram overlapping prognosis-associated PRGs independently screened from three separate NB cohorts to identify the single PRG (PARP1) with consistent prognostic significance across all datasets. **(B)** Differential expression of PARP1 in different survival states, different COG risks,and different MYCN amplification states of NB children in the GSE-NB cohort. **(C)** Expression of PARP1 in children with neuroblastoma in our hospital. **(a)** Inss stage 4,COG: High risk,MYCN:amplification,GIS:7,x400. **(b)** Inss stage 3,COG:moderate risk,MYCN:non-amplification,GIS:5,x400. **(c)** Inss stage 2,COG:moderate risk,MYCN:unknown,GIS:4,x400. **(d)** Inss stage 1,COG:low risk,MYCN:non-amplification,GIS:2,x400. **(D)** A nomogram for predicting NB prognosis. **(E)** The calibration curve, ROC curve,and DCA curve of the prediction model. ****p<0.0001.

Immunohistochemical staining was performed on tumor samples collected from 127 pediatric NB patients to quantify endogenous PARP1 expression. We found that the expression of PARP1 was different in pathologic sections of NB with different COG risk levels, MYCN amplification status and pathological stages, which may be related to prognosis. We converted the mean value of the staining intensity into a GIS score, with 0-3 as a low expression and 4-7 as a high expression. The distribution of PARP1 staining intensity is shown in (**Figure 3C**).

The expression level of PARP1 in 127 NB clinical samples was quantified by GIS score, and the GIS score was divided into two groups: the high expression group and the low expression group. COX regression analysis was performed based on the age, sex, MYCN amplification status, INSS stage, COG risk and other critical clinical information of the children. The results showed that MYCN status, COG risk and GIS score were correlated with OS in NB children, and MYCN amplification status and GIS score were independent prognostic factors affecting NB children (**Table 1**). Nomograms were drawn according to the results of multivariate COX regression analysis, which showed that MYCN amplification status and GIS score were indeed independent risk factors affecting OS in children with NB, and the higher the GIS score, the worse the prognosis of children **Figure 3D**). The C index of the prediction model was 0.743 (95%CI 0.670-0.815), Indicating that the prediction model of the column graph had good recognition ability. The OS of the predicted value of the nomogram prediction model is highly consistent with the actual observed value, indicating that the model has good accuracy. The AUC for 1, 3, and 5 years is 0.701, 0.703, and 0.627, respectively, suggesting that the nomogram is well discriminated. In addition, the DCA showed that the nomogram has good clinical application prospects, and compared with the traditional COG risk grouping, the nomogram model combining clinical information and the expression level of PARP1 obtained more net benefits (**Figure 3E**).

**Table 1:** Univariate and multivariate analyses of OS in our NB set.

Risk factors	Univariate			Multivariate		
	HR	95%CI	P	HR	95%CI	P
Age						
≤18months						
>18months	1.46	0.75-2.83	0.265			
Sex						
boy						
girl	0.76	0.39-1.47	0.409			
MYNC.status						
Non-amplification						
Amplified	5.92	2.79-12.55	<0.001	3.708	1.716-8.012	0.001
unknown	1.76	0.75-4.15	0.197	1.741	0.737-4.117	0.206
Inss.stage						
stage I						
stage II	1.94	0.12-31.01	0.64			
stage III	2.19	0.23-21.15	0.497			
stage IV	9.82	1.34-71.88	0.024			
stage IVs	11.32	1.03-125	0.048			
COG.risk						
low risk						
Mid risk	1.99	0.18-22.06	0.575			
high risk	11.54	1.58-84.4	0.016			
GIS score						
low risk						
high risk	2.75	1.26-5.99	0.011	2.64	1.199-5.813	0.016

### Inhibitory effect and safety evaluation *in vivo*

To evaluate the *in vivo* inhibitory effect of AZD5305, researchers administered two doses of AZD5305 to animals with neuroblastoma (NB), using cisplatin as the positive control. Continuous monitoring of subcutaneous tumor volume revealed that starting from day 5, the tumor volume in the AZD5305-treated group was significantly smaller than that in the control group, with the inhibitory effect increasing with higher doses. A dose of 1 mg/kg AZD5305 showed no significant tumor inhibition, with only a difference in tumor volume observed on day 7; in contrast, the 2 mg/kg dose of AZD5305 demonstrated pronounced *in vivo* inhibitory effects (**Figures 4A–D**). Additionally, tumors in the cisplatin-positive control group exhibited significantly slowed growth rates, markedly reduced tumor volumes, and substantial weight reduction, although this group also experienced notable weight loss in mice. When AZD5305 was combined with cisplatin, the AZD5305 + high-dose cisplatin combination group still showed significant tumor inhibition but was accompanied by marked weight loss in mice. In contrast, the AZD5305 + low-dose cisplatin group (2.5 mg/kg) similarly demonstrated significant slowing of tumor growth rate, marked tumor volume reduction, and substantial weight reduction, without significant weight loss. These results confirm that the AZD5305 combination therapy enhances therapeutic efficacy while minimizing cisplatin-induced toxic side effects. Additionally, tumor HE staining revealed significant reduction in cytoplasmic proteins following AZD5305 treatment, accompanied by intracellular vacuolation, localized hemorrhage, and necrosis of tumor cells (**Figure 4E**).

**Figure 4:**
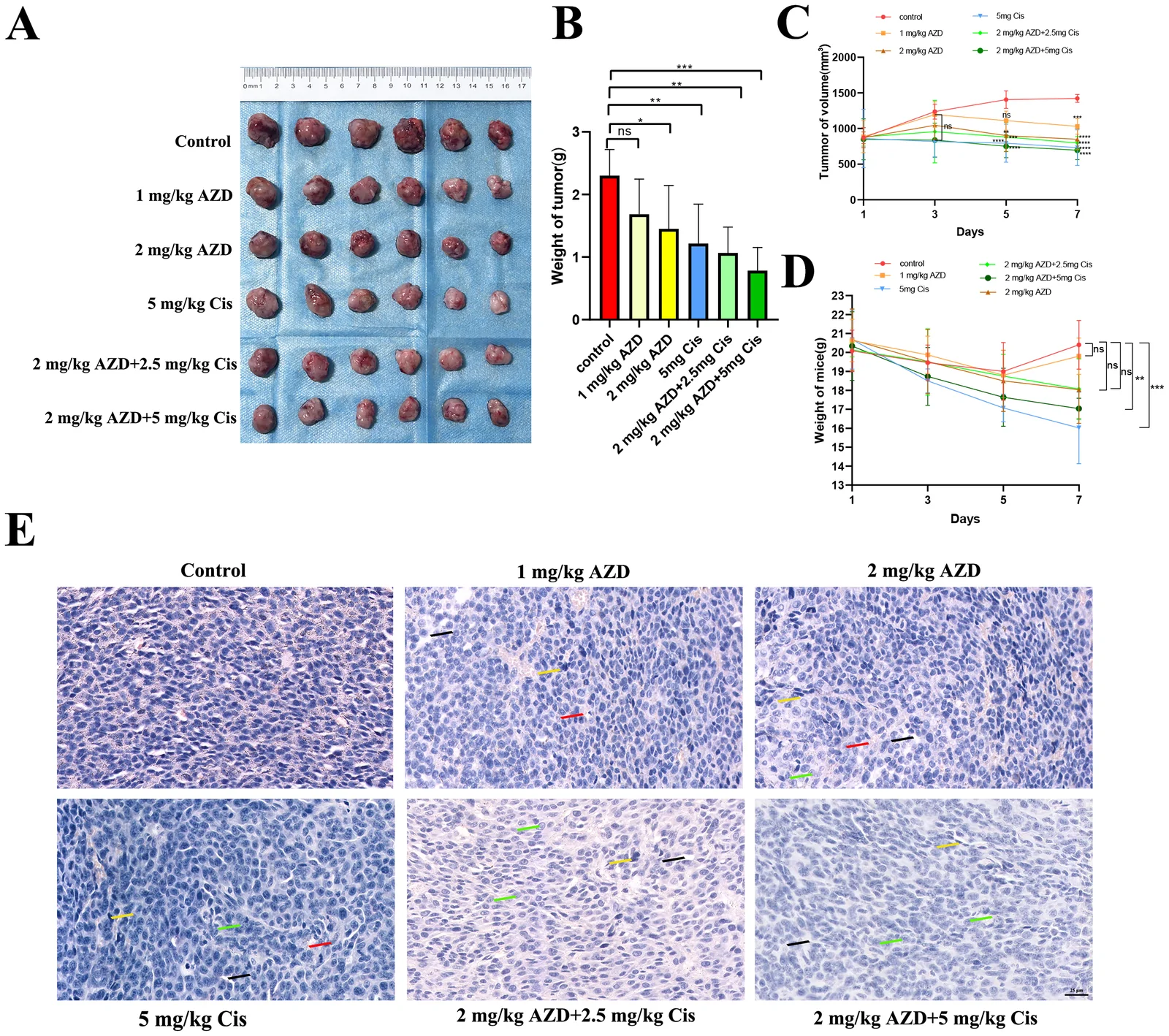
Evaluation of the therapeutic efficacy of AZD5305. **(A)** Tumor tissue. **(B)** Tumor weight. **(C)** Tumor growth curve. **(D)** Body weight of mice. **(E)** HE staining results of the tumor. White arrow: intercellular space; Yellow arrow: cellular necrosis; Red arrow: local hemorrhage. Scale bar: 25 μm. Compared with the control group:NS p> 0.05(), *p <0.05, **p <0.01, ***p <0.001.

Furthermore, serum biochemical indicators in mice demonstrated that AZD5305 alone did not cause significant hepatorenal injury, whereas the cisplatin group exhibited elevated levels of CREA, BUN, ALT, and AST. In contrast, the combination of AZD5305 and cisplatin markedly reduced these parameters, indicating mitigated hepatic and renal damage (**Figure 5A**). HE staining revealed no significant differences among the groups in the heart, spleen, and lungs. In renal tissues, the AZD5305 monotherapy group showed no notable renal injury compared to the cisplatin group; the combined AZD5305 and low-dose cisplatin regimen further alleviated structural damage in renal tissues, reduced vacuolization of tubular epithelial cells, decreased cellular swelling, necrosis, and detachment, and diminished inflammatory cell infiltration. Mice in the cisplatin group exhibited hepatic tissue injury, marked cellular swelling, hepatic sinusoidal congestion, and focal infiltration of numerous inflammatory cells. In contrast, the AZD5305 monotherapy group showed no significant liver damage. The combination therapy group demonstrated reduced hepatocyte swelling, improved disordered hepatocyte arrangement, alleviated hepatic sinusoidal congestion and venous dilation, as well as decreased inflammatory cell infiltration in the liver parenchyma and perivascular areas (**Figure 5B**). The Masson trichrome staining results of renal tissues demonstrated significant collagen deposition in the kidney tissues of mice treated with cisplatin, whereas no notable collagen deposition was observed in the AZD5305 monotherapy group. The combination therapy with AZD and cisplatin further reduced collagen deposition, indicating that cisplatin induces renal fibrosis, while the AZD5305 regimen mitigates this effect (**Figure 5C**).

**Figure 5:**
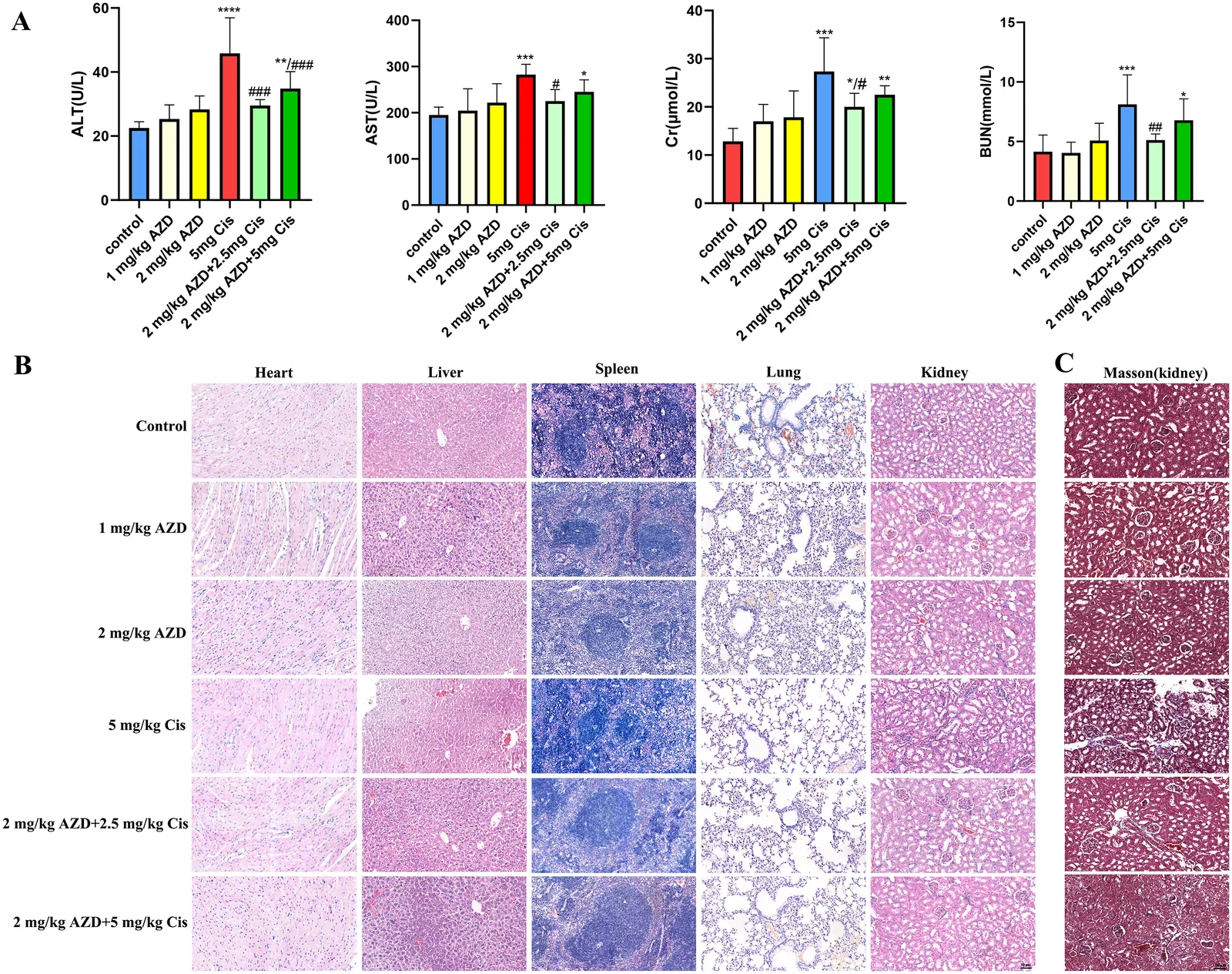
Evaluation of the safety of AZD5305. **(A)** ALT, AST, Cr and BUN. **(B)** HE Staining of heart, liver, kinder, lung and spleen, Scale bar: 50μm. **(C)** Marson tricolor staining of renal tissue. * present Compared with the control group, p <0.05; **present Compared with the control group,p <0.01; ***present Compared with the control group, p <0.001; ****present Compared with the control group, p <0.0001; ^#^present Compared with the 5mg Cis group,p<0.05; ^##^present Compared with the 5mg Cis group,p<0.01; ^####^present Compared with the 5mg Cis group,p<0.001.

### Cytotoxicity of AZD5303

The CCK8 assay demonstrated that after 48-hour treatment with AZD5305, the IC50 for SK-N-SH and SK-N-DZ cells were 84 µM and 114 µM respectively (**Figures 6A, D**). Subsequent treatments with low, medium, and high doses (1/2 IC50, IC50, and 2 IC50) showed a dose-dependent decrease in proliferative activity compared to the control group (**Figures 6B, E**). Edu cell staining revealed significantly reduced proliferation capacity in the AZD5305-treated cells (**Figures 6C, F**).

**Figure 6:**
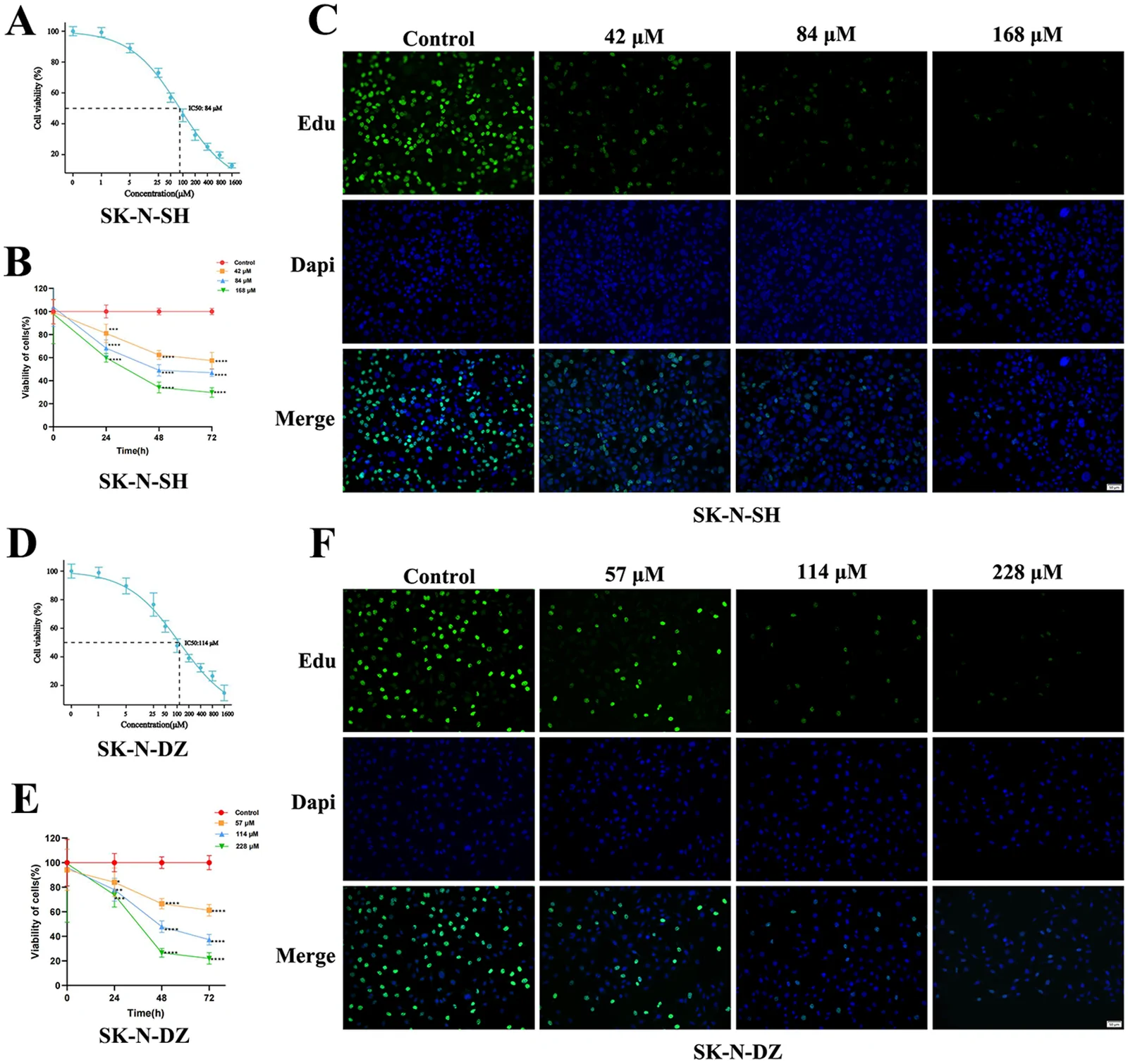
Cytotoxicity of AZD5305. **(A, D)** IC50 values of AZD5305 on SK-N-SH and SK-N-DZ. **(B, E)** Cell proliferation curves of SK-N-SH and SK-N-DZ. **(C, F)** Edu assays on SK-N-SH and SK-N-DZ. Scale bar: 50 μm. *p< 0.05; **p <0.01; ***p <0.001; ****p <0.0001.

### AZD5303 inhibited cell migration and invasion

The wound healing experiments demonstrated that SK-N-SH and SK-N-DZ cells in the AZD-treated group showed significantly reduced migration rates at 24 and 48 hours post-treatment compared to the control group (**Figures 7A, B**). Transwell assays performed 48 hours after AZD treatment further revealed a marked decrease in cell penetration through the matrix gel, indicating that AZD5305 effectively inhibits the invasive capacity of neuroblastoma cells (**Figures 7C, D**).

**Figure 7:**
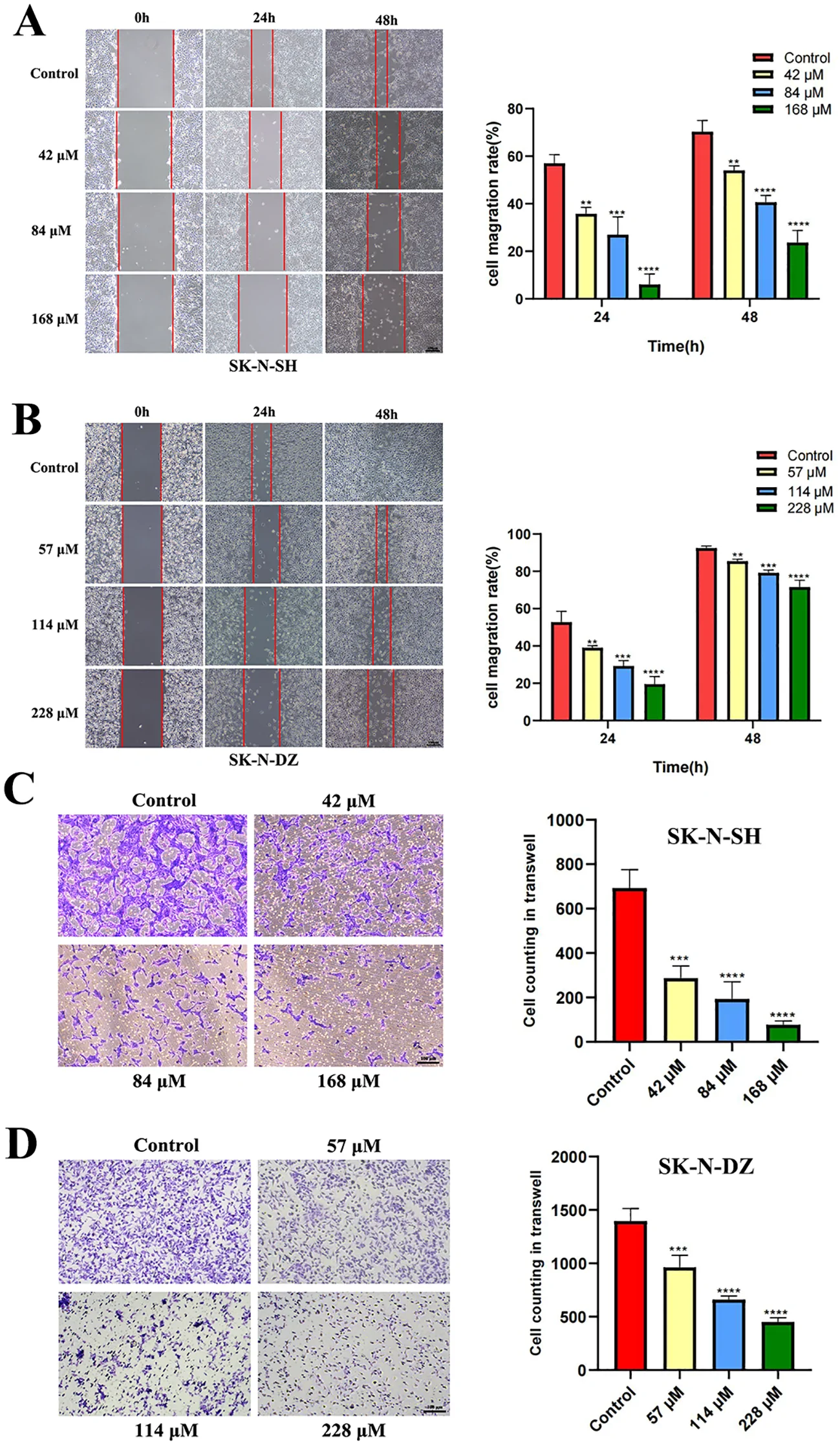
AZD5305 inhibits cell migration and invasion. **(A, B)** Wound healing assay of SK-N-SH and SK-N-DZ. **(C, D)** Transwell assay of SK-N-SH and SK-N-DZ. Scale bar: 100 μm. **p <0.01; ***p <0.001; ****p <0.0001.

### AZD5305 induces apoptosis and G0/G1 Arrest

The Annexin-V apoptosis assay demonstrated a significant increase in cell death levels following AZD5305 treatment (**Figures 8A, C**). The apoptosis response showed distinct differences between the two cell lines, with SK-N-SH cells exhibiting heightened sensitivity to apoptosis. Flow cytometry analysis revealed that both NB cell lines experienced a marked increase in G0/G1 phase cells after AZD5305 treatment (**Figures 8B, D**).

**Figure 8:**
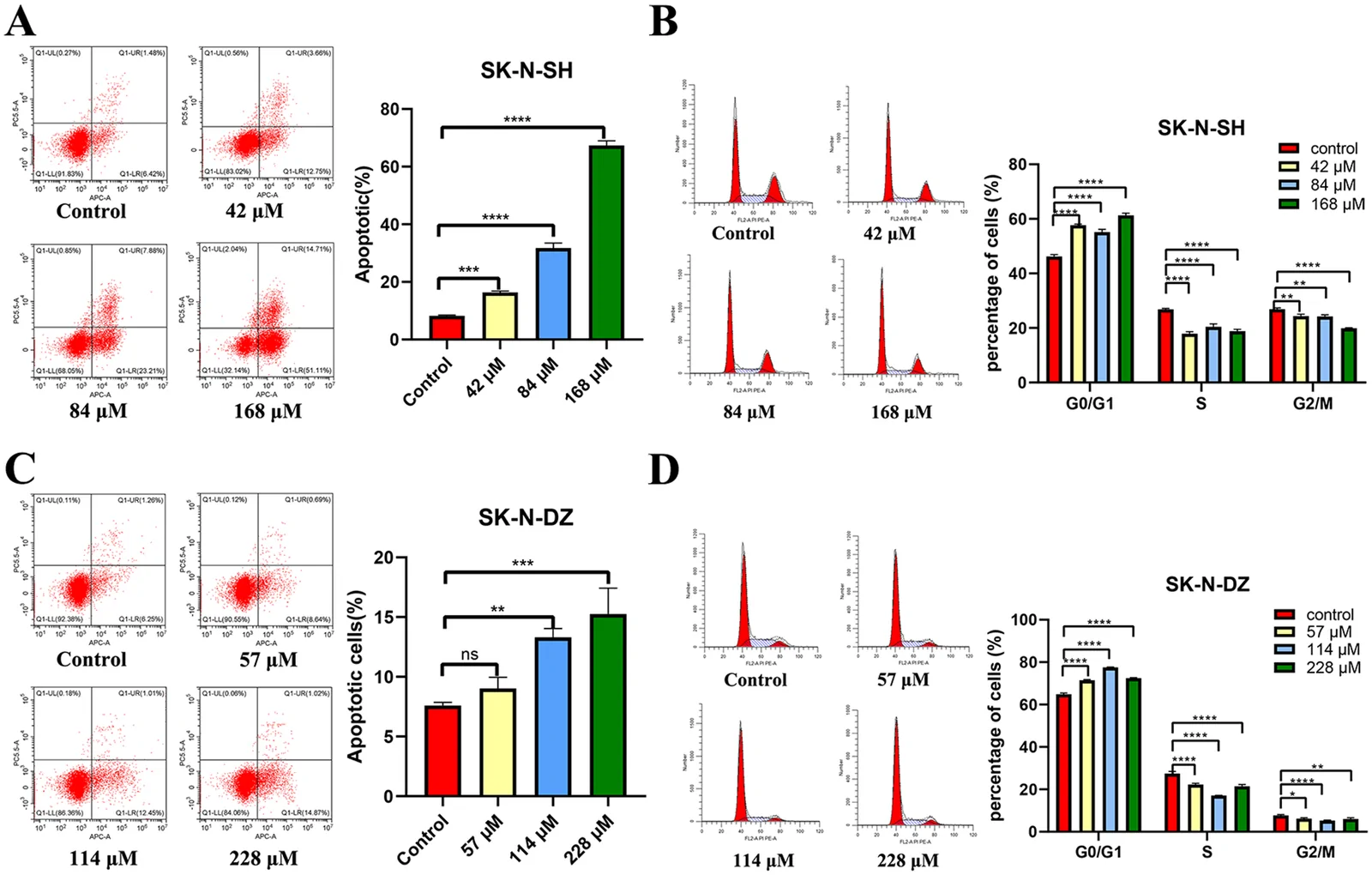
AZD5305 induces apoptosis and G0/G1 arrest. **(A, C)** Annexin V apoptosis assay in SK-N-SH and SK-N-DZ. **(B, D)** Cell cycle of SK-N-SH and SK-N-DZ. ns p>0.05, *p< 0.05; **p <0.01; ***p <0.001; ****p <0.0001.

## Discussion

Neuroblastoma is a high-mortality pediatric extracranial solid tumor with limited effective targeted therapies. PANoptosis integrates pyroptosis, apoptosis and necroptosis and plays vital roles in tumor progression and immune microenvironment remodeling. In this work, we systematically analyzed PANoptosis-related genes (PRGs) across three independent NB datasets, screened the shared prognostic gene PARP1, and verified the anti-tumor activity of the PARP1 selective inhibitor AZD5305 via *in vitro* and *in vivo* experiments. Different from conventional prognostic biomarker studies that construct a fixed signature in one cohort and validate its predictive ability in external datasets, our study adopted a three-cohort parallel independent screening strategy to mine conserved therapeutic targets stably correlated with NB prognosis.

To date, substantial evidence suggests that PCD subroutines, including apoptosis, pro-death, and necrosis, play a key role in tumorigenesis; loss of control over a single or combined PCD subroutine may contribute to cancer development (6). While PANoptosis has critical features of both apoptosis, pro-death, and necrosis pathways, it cannot be explained by any of them alone. PANoptosis maintains homeostasis through “PANoptosome”. Therefore, significant crosstalk occurs in the execution and regulation of numerous pathways in PANoptosis (30). Many PRGs are abnormally expressed in various cancer types, and significant mutations have been detected. Notably, many PRGs have been confirmed as tumour risk factors in different types of cancer, such as ZBP1, MAP3K7 and RBCK1, which have been identified as associated with OS in cutaneous melanoma and can be used as reliable prognostic biomarkers for cutaneous melanoma (9). The expression levels of seven PRGs, including CARD16, LAP3, CCR7, VSIG4, LGR5, GZMB and ITLN1, have been confirmed to be associated with the malignant progression of colon cancer (10). The survival of tumour cells is closely related to the fact that the tumour microenvironment in which they are located helps them escape immune surveillance and drug interference (31). We evaluated the PANoptotic signature and immune infiltration relationship in children with NB using the TME algorithm, and we observed relatively high stromal scores, immune scores, and estimated scores in the low-risk group of PANpoptotic cells. Significant differences in immune cell infiltration existed between the high-risk and low-risk groups for PANoptosis. In addition, our results also showed that the risk score of PANpoptosis was significantly correlated with the abundance of immune cells, and it was observed that the abundance of M2 macrophages was positively correlated with the risk score of PANoptosis. Wang et al. also observed that the abundance of M2 macrophages was positively correlated with the risk score of PANoptosis in colon cancer (10). It is suggested that the future selection and efficacy evaluation of immunotherapy may be related to the risk score of PANoptosis in cancer. This study characterized 19 PRGs reported in previous literature in NB. Subsequently, LASSO and multifactor Cox regression analysis were used to screen PRGs to construct prognostic characteristics. In this study, the GSE49711-NB cohort was used as the primary study object, and 11 PRGs were found to be closely related to the prognosis of NB. They were PARP1, FADD, TRADD, CASP8, MLKL, AIM2, TAB3, TNFAIP3, IRF1, PSTPIP2 and CASP7; 5 PRGs, PARP1, TAB2, MLKL, ZBP1 and RIPK3, which were closely related to the prognosis of NB, were screened out in the Target-NB cohort. A total of 6 PRGs associated with NB prognosis were identified in the E-MTAB-8248-NB cohort, namely PARP1, RIPK1, GSDMD3, CASP7, FADD and NLRP3. By intersecting the results of the three independent cohorts, we screened out the key PRGs: PARP1, which was associated with the prognosis of NB. PARP (Poly ADP-ribose polymerase) is a DNA repair enzyme encoded by the PARP gene and plays a vital role in DNA damage repair and genome stability maintenance in humans. Genomic instability is one of the characteristics of tumorigenesis. In order to maintain genomic integrity, cells use a variety of repair mechanisms to repair a variety of DNA damage caused by exogenous or endogenous events. The function of PARP is to bind to DNA damage sites (mostly single-strand DNA breaks) and catalyze the synthesis of polyADP ribose chains on protein substrates to repair DNA damage by recruiting DNA repair proteins to the damage sites (32). PARP1 is one of the critical genes in the 19 PRGs reported so far. As the most essential member of the PARP family, PARP1 carries more than 90% of the functions of the PARP family in cells and is a critical factor in DNA damage repair (33). PARP1 was previously reported to be differentially expressed in NB cell lines and associated with genetic heterogeneity in children with NB (34). However, the prognostic effect of PARP1 on NB and how it affects NB’s progression have not been reported. According to the results of COX regression analysis, PARP1 was an independent risk factor associated with NB and prognosis in all three NB cohorts. The HR of PARP1 in the GSE49711-NB cohort was 4.36 (95%CI: 2.50-7.60), the HR of PARP1 in the Target-NB cohort was 1.92 (95%CI: 1.27-2.90), and the HR of PARP1 in E-MTAB-8248-NB cohort was 5.88 (95%CI: 2.88-12.35). These results indicate that the higher the expression of PARP1, the worse the prognosis of NB patients. We further collected clinical specimens of NB children and detected the expression level of PARP1 in NB samples by immunohistochemistry. The results showed that PARP1 expression level significantly differed in different Inss stages, MYCN amplification status and COG risk groups. Subsequently, we conducted a multivariate COX regression analysis combining PARP1 expression with clinical information on children with NB, and we found that the PARP1 expression level was significantly associated with the prognosis of children with NB. The expression level of PARP1 is significantly correlated with the prognosis of NB children, suggesting that the treatment of PARP1 may become one of the potential strategies for NB. Marianna Avitabile et al. found that functional single nucleotide polymorphisms (SNPs) of PARP1 can predict the response of high-risk NB patients to current induction therapy (35). The study of V Colicchia et al. also found that we found that the high expression of PARP1 and PARP2 was significantly correlated with advanced, high-risk NB cases. High expression of PARP1 and PARP2 (mainly PARP1) is significantly associated with high-risk NB cases and low survival, suggesting that it is a potential and previously unrecognized prognostic factor for human NB (36). PARP inhibitors are small molecule targeted drugs based on a synergistic synthesis of lethal effects. By binding to the PARP1 or PARP2 catalytic sites, PARP inhibitors cause the PARP protein to be unable to be shed from the DNA damage site. PRAP bound to DNA will lead to the arrest of DNA replication forks during DNA replication, which leads to the failure of DNA replication. Normal cells can repair DNA double-strand damage through a homologous recombination repair mechanism. In contrast, the DNA of BRCA-deficient (HR deficient) tumour cells can not be repaired in time and transformed into a fatal double-strand break, thus killing tumour cells (37). Since the launch of the first PARP inhibitor in 2014, six PARP inhibitors have been introduced worldwide. The successful research and development of PARP inhibitors have brought revolutionary changes to the drug treatment of some tumours, especially in the field of ovarian cancer, making the drug treatment of recurrent ovarian cancer enter an era of precision therapy (38). In addition, the therapeutic effects of PARP inhibitors on breast cancer, fallopian tube cancer, pancreatic cancer and prostate cancer have been gradually discovered and applied to clinical practice (39), which is expected to become a broad-spectrum anti-tumour drug. Therefore, the research and development of PARP inhibitors has become a hot spot in the field of anticancer. So far, PARP inhibitors have achieved encouraging results in preclinical models of NB. However, previous studies have focused on the enhancement or synergistic effect of PARP inhibitors on the anti-tumor effects of other drugs. There is no reference to the expression of the PARP1 gene in NB after using PARP1 inhibitors, nor to the effects and specific mechanisms of PARP1 inhibitor alone on NB (40–42).

As a selective PARP1 inhibitor, AZD5303 is superior to the first-generation PARPi in selecting PARP1. Giuditta Illuzzi et al. found that mice treated with AZD5305 continued to benefit from anti-tumor effects, and their tumor response lasted for more than 100 days (43). AZD5305 is currently undergoing Phase I clinical trials (NCT04644068). Research by Andrea Herencia-Ropero et al. has also demonstrated that AZD5305 exhibits superior antitumor activity compared to first-generation PARPi in patient-derived BRCA1/2-related cancer models, with a complete response rate of 75% versus 37% (44). Studies have also found that the combination of AZD5305 and platinum-based drugs can inhibit the spread of tumor metastases and significantly prolong the survival of mice with abdominal ovarian cancer primary xenografts (OC-PDX), this synergistic effect remains significant even when cisplatin monotherapy is ineffective (45). However, no studies have been reported on the role of AZD5305 in NB, so we used AZD5303 for functional experiments. The results showed that AZD5305 inhibited the proliferation, migration and invasion of neuroblastoma cells, induced apoptosis and G0/G1 arrest.AZD5305 inhibits the growth of neuroblastoma, and its single administration does not cause significant organ damage. Notably, the combination of AZD5305 and low-dose cisplatin achieved an ideal therapeutic balance between efficacy and safety. AZD5305 not only synergistically enhanced the anti-tumor effect of cisplatin, but also relieved chemotherapy-induced organ damage and renal fibrosis, which provides a feasible solution to the dilemma of high-dose chemotherapy in pediatric neuroblastoma.

## Limitations

Of course, this study has some limitations. First, the data analyzed in this study came from public databases and were all retrospective studies, lacking random prospective samples. These public neuroblastoma cohorts exhibit inherent inter-cohort heterogeneity, including differences in sequencing platforms, sample sizes, and distributions of clinical characteristics. Our current analytical framework prioritizes screening cross-cohort stable therapeutic targets rather than constructing a unified predictive signature. Although our multi-cohort parallel intersection strategy helps filter out biased markers only applicable to a single dataset and the prognostic value of PARP1 has been further validated using clinical specimens from our institution, prospective multicenter studies are still required to facilitate clinical translation. For future research focused on prognostic risk scoring, we will adopt the standard discovery-validation pipeline recommended by reviewers to fix signature genes and verify their predictive performance in external cohorts. Second, although PARP1 was retrieved from published PANoptosis-related gene sets and identified as a shared prognostic gene across the three cohorts, our existing data cannot confirm whether PARP1 directly governs the complete PANoptosis signaling cascade in neuroblastoma. PARP1 can serve as an independent prognostic biomarker and actionable therapeutic target, and its regulatory role in PANoptosis awaits dedicated follow-up investigations. Third, although we have observed that AZD5305 inhibits tumor proliferation and migration, induces G0/G1-phase cell-cycle arrest and triggers apoptosis both *in vitro* and *in vivo*, the exact downstream signaling events remain incompletely elucidated. Core DNA-damage-response molecules including γ-H2AX, p-RPA2, PARylation products, p-ATR, p-CHK1, RAD51 and p21 were not examined in the present work. To address this mechanistic gap, AZD5305-treated SK-N-SH and SK-N-DZ cells have been submitted for transcriptome sequencing, and we will further explore the mechanisms by which AZD5305 modulates the PANoptosis cascade to suppress neuroblastoma progression in future work.

Nevertheless, our study is still the first to perform a PANoptosis-related analysis in pediatric NB. We cross-referenced results from three public databases to screen out essential genes and validated our findings using clinical samples and clinical information from our hospital. In brief, combined bioinformatic and experimental validation suggests that PANoptosis is associated with survival outcomes of children with NB, and key PRGs are identified as potential markers for diagnosis and treatment. Furthermore, preliminary findings from *in vivo* and *in vitro* experiments demonstrate that AZD5305 inhibits neuroblastoma growth by promoting apoptosis and inducing cell-cycle arrest. Additionally, its combination with cisplatin mitigates cisplatin-induced weight loss and hepatic/renal function impairment. In particular, our findings pioneer the application of AZD5305 in neuroblastoma treatment and firmly support this selective PARP1 inhibitor as a promising novel targeted therapy for children with refractory neuroblastoma.

## Data Availability

The original contributions presented in the study are included in the article/**Supplementary Material**. Further inquiries can be directed to the corresponding author.

## Supplementary material

Supplementary Figure 1 Identification and comprehensive analysis of PANoptotic subtypes in the GSE-NB cohort. **(A)** Spearman correlation analysis confirmed that the expression of 19 PRGs in the GSE-NB cohort was correlated to varying degrees. **(B)** Cluster analysis of two subgroups C1 and C2. **(C)** Principal component analysis(PCA)of two subgroups C1 and C2. **(D)** The KM curve shows the survival difference between the two subgroups C1 and C2. **(E)** Heat map showing clinicopathological features and associations of 19 PRGs between the C1 and C2 subgroups. **(F)** Matrix scores,immune scores,and estimated scores observed in the C1 and C2 subtypes of the GSE49711 cohort. **(G)** Infiltration of immune cells in the C1 and C2 subtypes. (H)GSVA enrichment analysis of C1 and C2 subtypes.

## References

[1] Schnepp RW Khurana P Attiyeh EF Raman P Chodosh SE Oldridge DA . A LIN28B-RAN-AURKA signaling network promotes neuroblastoma tumorigenesis. Cancer Cell. (2015) 28:599–609. doi: 10.1016/j.ccell.2015.09.012 26481147 PMC4643330

[2] De Bernardi B Quaglietta L Haupt R Castellano A Tirtei E Luksch R . Neuroblastoma with symptomatic epidural compression in the infant: the AIEOP experience. Pediatr Blood Cancer. (2014) 61:1369–75. doi: 10.1002/pbc.25028 24619960

[3] London WB Castel V Monclair T Ambros PF Pearson AD Cohn SL . Clinical and biologic features predictive of survival after relapse of neuroblastoma: a report from the International Neuroblastoma Risk Group project. J Clin Oncol. (2011) 29:3286–92. doi: 10.1200/jco.2010.34.3392 21768459 PMC3158599

[4] Berlanga P Canete A Castel V . Advances in emerging drugs for the treatment of neuroblastoma. Expert Opin Emerg Drugs. (2017) 22:63–75. doi: 10.1080/14728214.2017.1294159 28253830

[5] Kopeina GS Zhivotovsky B . Programmed cell death: past, present and future. Biochem Biophys Res Commun. (2022) 633:55–8. doi: 10.1016/j.bbrc.2022.09.022 36344162

[6] Peng F Liao M Qin R Zhu S Peng C Fu L . Regulated cell death (RCD) in cancer: key pathways and targeted therapies. Signal Transduct Target Ther. (2022) 7:286. doi: 10.1038/s41392-022-01110-y 35963853 PMC9376115

[7] Wang Y Kanneganti TD . From pyroptosis, apoptosis and necroptosis to PANoptosis: a mechanistic compendium of programmed cell death pathways. Comput Struct Biotechnol J. (2021) 19:4641–57. doi: 10.1016/j.csbj.2021.07.038 34504660 PMC8405902

[8] Zhang Z Zhang F Pang P Li Y Chen X Sun S . Identification of PANoptosis-relevant subgroups to evaluate the prognosis and immune landscape of patients with liver hepatocellular carcinoma. Front Cell Dev Biol. (2023) 11:1210456. doi: 10.3389/fcell.2023.1210456 37325556 PMC10267832

[9] Wang W Zhou Q Lan L Xu X . PANoptosis-related prognostic signature predicts overall survival of cutaneous melanoma and provides insights into immune infiltration landscape. Sci Rep. (2023) 13:8449. doi: 10.1038/s41598-023-35462-4 37231081 PMC10213036

[10] Wang X Sun R Chan S Meng L Xu Y Zuo X . PANoptosis-based molecular clustering and prognostic signature predicts patient survival and immune landscape in colon cancer. Front Genet. (2022) 13:955355. doi: 10.3389/fgene.2022.955355 36186438 PMC9515384

[11] Zhuang L Sun Q Huang S Hu L Chen Q . A comprehensive analysis of PANoptosome to prognosis and immunotherapy response in pan-cancer. Sci Rep. (2023) 13:3877. doi: 10.1038/s41598-023-30934-z 36890219 PMC9995449

[12] Karki R Sundaram B Sharma BR Lee S Malireddi RKS Nguyen LN . ADAR1 restricts ZBP1-mediated immune response and PANoptosis to promote tumorigenesis. Cell Rep. (2021) 37:109858. doi: 10.1016/j.celrep.2021.109858 34686350 PMC8853634

[13] Gao J Xiong A Liu J Li X Wang J Zhang L . PANoptosis: bridging apoptosis, pyroptosis, and necroptosis in cancer progression and treatment. Cancer Gene Ther. (2024) 31:970–83. doi: 10.1038/s41417-024-00765-9 38553639 PMC11257964

[14] Zhao K Wang X Jin Y Zhu X Zhou T Yu Y . LncRNA ZNF674-AS1 drives cell growth and inhibits cisplatin-induced pyroptosis via up-regulating CA9 in neuroblastoma. Cell Death Dis. (2024) 15:5. doi: 10.1038/s41419-023-06394-8 38177154 PMC10766958

[15] Li W Li X Xia Y Wang J . Pyroptosis-related gene signature predicts the prognosis and immune infiltration in neuroblastoma. Front Genet. (2022) 13:809587. doi: 10.3389/fgene.2022.809587 35664308 PMC9162481

[16] Luo B Wang L Gao W Su Y Lu Y Zheng J . Using a gene network of pyroptosis to quantify the responses to immunotherapy and prognosis for neuroblastoma patients. Front Immunol. (2022) 13:845757. doi: 10.3389/fimmu.2022.845757 35401536 PMC8987018

[17] Liu G Pei F Yang F Li L Amin AD Liu S . Role of autophagy and apoptosis in non-small-cell lung cancer. Int J Mol Sci. (2017) 18(4):430–5. doi: 10.3390/ijms18020367 28208579 PMC5343902

[18] Fulda S . Apoptosis pathways and neuroblastoma therapy. Curr Pharm Des. (2009) 15:430–5. doi: 10.2174/138161209787315846 19199970

[19] Nomura M Ueno A Saga K Fukuzawa M Kaneda Y . Accumulation of cytosolic calcium induces necroptotic cell death in human neuroblastoma. Cancer Res. (2014) 74:1056–66. doi: 10.1158/0008-5472.can-13-1283 24371227

[20] Maurer BJ Metelitsa LS Seeger RC Cabot MC Reynolds CP . Increase of ceramide and induction of mixed apoptosis/necrosis by N-(4-hydroxyphenyl)-retinamide in neuroblastoma cell lines. J Natl Cancer Inst. (1999) 91:1138–46. doi: 10.1093/jnci/91.13.1138 10393722

[21] Nguyen LN Kanneganti TD . PANoptosis in viral infection: the missing puzzle piece in the cell death field. J Mol Biol. (2022) 434:167249. doi: 10.1016/j.jmb.2021.167249 34537233 PMC8444475

[22] Wilkerson MD Hayes DN . ConsensusClusterPlus: a class discovery tool with confidence assessments and item tracking. Bioinformatics. (2010) 26:1572–3. doi: 10.1093/bioinformatics/btq170 20427518 PMC2881355

[23] Hanzelmann S Castelo R Guinney J . GSVA: gene set variation analysis for microarray and RNA-seq data. BMC Bioinf. (2013) 14:7. doi: 10.1093/bioinformatics/btq170 PMC3618321 23323831

[24] Liberzon A Subramanian A Pinchback R Thorvaldsdóttir H Tamayo P Mesirov JP . Molecular signatures database (MSigDB) 3.0. Bioinformatics. (2011) 27:1739–40. doi: 10.1093/bioinformatics/btr260 21546393 PMC3106198

[25] Ritchie ME Phipson B Wu D Hu Y Law CW Shi W . limma powers differential expression analyses for RNA-sequencing and microarray studies. Nucleic Acids Res. (2015) 43:e47. doi: 10.1093/nar/gkv007 25605792 PMC4402510

[26] Yoshihara K Shahmoradgoli M Martínez E Vegesna R Kim H Torres-Garcia W . Inferring tumour purity and stromal and immune cell admixture from expression data. Nat Commun. (2013) 4:2612. doi: 10.1038/ncomms3612 24113773 PMC3826632

[27] Tian XM Xiang B Yu YH Li Q Zhang ZX Zhanghuang C . A novel cuproptosis-related subtypes and gene signature associates with immunophenotype and predicts prognosis accurately in neuroblastoma. Front Immunol. (2022) 13:999849. doi: 10.3389/fimmu.2022.999849 36211401 PMC9540510

[28] Liu T Zhu C Chen X Wu J Guan G Zou C . Dual role of ARPC1B in regulating the network between tumor-associated macrophages and tumor cells in glioblastoma. Oncoimmunology. (2022) 11:2031499. doi: 10.1080/2162402x.2022.2031499 35111386 PMC8803105

[29] Jin L Mi T Wu X Wang Z Zhang Z Liu J . BI-D1870 induces mitotic dysfunction and apoptosis in neuroblastoma by regulating the PI3K-Akt-mTORC1 signal axis. Cancers (Basel). (2023) 15:2023. doi: 10.3390/cancers15072023 37046682 PMC10093276

[30] Samir P Malireddi RKS Kanneganti TD . The PANoptosome: a deadly protein complex driving pyroptosis, apoptosis, and necroptosis (PANoptosis). Front Cell Infect Microbiol. (2020) 10:238. doi: 10.3389/fcimb.2020.00238 32582562 PMC7283380

[31] Argilés JM López-Soriano FJ Stemmler B Busquets S . Cancer-associated cachexia-understanding the tumour macroenvironment and microenvironment to improve management. Nat Rev Clin Oncol. (2023) 20:250–64. doi: 10.3389/fcimb.2020.00238 36806788

[32] Cao C Yang J Chen Y Zhou P Wang Y Du W . Discovery of SK-575 as a highly potent and efficacious proteolysis-targeting chimera degrader of PARP1 for treating cancers. J Med Chem. (2020) 63:11012–33. doi: 10.1021/acs.jmedchem.0c00821 32924477

[33] Peng X Pan W Jiang F Chen W Qi Z Peng W . Selective PARP1 inhibitors, PARP1-based dual-target inhibitors, PROTAC PARP1 degraders, and prodrugs of PARP1 inhibitors for cancer therapy. Pharmacol Res. (2022) 186:106529. doi: 10.1016/j.phrs.2022.106529 36328301

[34] Cheng J Zhuo Z Zhao P Zhu J Xin Y Zhang J . PARP1 gene polymorphisms and neuroblastoma susceptibility in Chinese children. J Cancer. (2019) 10:4159–64. doi: 10.7150/jca.34222 31413734 PMC6691706

[35] Avitabile M Lasorsa VA Cantalupo S Cardinale A Cimmino F Montella A . Association of PARP1 polymorphisms with response to chemotherapy in patients with high-risk neuroblastoma. J Cell Mol Med. (2020) 24:4072–81. doi: 10.1111/jcmm.15058 32103589 PMC7171401

[36] Colicchia V Petroni M Guarguaglini G Sardina F Sahún-Roncero M Carbonari M . PARP inhibitors enhance replication stress and cause mitotic catastrophe in MYCN-dependent neuroblastoma. Oncogene. (2017) 36:4682–91. doi: 10.1038/onc.2017.40 28394338

[37] Fontaine SD Ashley GW Houghton PJ Kurmasheva RT Diolaiti M Ashworth A . A very long-acting PARP inhibitor suppresses cancer cell growth in DNA repair-deficient tumor models. Cancer Res. (2021) 81:1076–86. doi: 10.1158/0008-5472.can-20-1741 33323380 PMC8670222

[38] Wu X Zhu J Wang J Lin Z Yin R Sun W . Pamiparib monotherapy for patients with germline BRCA1/2-mutated ovarian cancer previously treated with at least two lines of chemotherapy: a multicenter, open-label, phase II study. Clin Cancer Res. (2022) 28:653–61. doi: 10.1158/0008-5472.can-20-1741 PMC9377729 34844979

[39] Kim C Wang XD Yu Y . PARP1 inhibitors trigger innate immunity via PARP1 trapping-induced DNA damage response. Elife. (2020) 9(4):653–61. doi: 10.7554/elife.60637 32844745 PMC7486119

[40] Daniel RA Rozanska AL Thomas HD Mulligan EA Drew Y Castelbuono DJ . Inhibition of poly(ADP-ribose) polymerase-1 enhances temozolomide and topotecan activity against childhood neuroblastoma. Clin Cancer Res. (2009) 15:1241–9. doi: 10.1158/1078-0432.ccr-08-1095 19174487

[41] McNeil EM Ritchie AM Melton DW . The toxicity of nitrofuran compounds on melanoma and neuroblastoma cells is enhanced by Olaparib and ameliorated by melanin pigment. DNA Repair (Amst). (2013) 12:1000–6. doi: 10.1016/j.dnarep.2013.08.017 24070777

[42] Southgate HED Chen L Tweddle DA Curtin NJ . Poly(ADP-ribose) polymerase inhibitor MK-4827 together with radiation as a novel therapy for metastatic neuroblastoma. Anticancer Res. (2013) 33:755–62. doi: 10.1016/j.dnarep.2013.08.017 PMC3684561 23482742

[43] Illuzzi G Staniszewska AD Gill SJ Pike A McWilliams L Critchlow SE . Preclinical characterization of AZD5305, a next-generation, highly selective PARP1 inhibitor and trapper. Clin Cancer Res. (2022) 28:4724–36. doi: 10.1158/1078-0432.ccr-22-0301 35929986 PMC9623235

[44] Herencia-Ropero A Llop-Guevara A Staniszewska AD Domènech-Vivó J García-Galea E Moles-Fernández A . The PARP1 selective inhibitor saruparib (AZD5305) elicits potent and durable antitumor activity in patient-derived BRCA1/2-associated cancer models. Genome Med. (2024) 16:107. doi: 10.1186/s13073-024-01370-z 39187844 PMC11348616

[45] Dellavedova G Decio A Formenti L Albertella MR Wilson J Staniszewska AD . The PARP1 inhibitor AZD5305 impairs ovarian adenocarcinoma progression and visceral metastases in patient-derived xenografts alone and in combination with carboplatin. Cancer Res Commun. (2023) 3:489–500. doi: 10.1158/2767-9764.crc-22-0423 36994441 PMC10042207

